# Determinants of neonatal jaundice among neonates admitted to neonatal intensive care unit in public hospitals of Sidama Region, Sidama, Ethiopia, 2022: an unmatched case-control study

**DOI:** 10.11604/pamj.2023.45.117.40472

**Published:** 2023-07-06

**Authors:** Biniyam Demessie Sisay, Rekiku Fikre Abebe, Ayalnesh Asmamaw Kassie, Melkamu Getu Wondimu, Gizachew Ambaw Kassie

**Affiliations:** 1 Department of Midwifery, Hawassa University, Hawassa, Sidama, Ethiopia,; 2 Department of Midwifery, Jimma University, Jimma, Oromia, Ethiopia,; 3 Department of Epidemiology and Biostatistics, School of Public Health, Wolayita Sodo University, Sodo, Ethiopia

**Keywords:** Neonatal jaundice, determinants, case-control study, Sidama region, public, hospitals

## Abstract

**Introduction:**

neonatal jaundice appears in most neonates as “physiological jaundice” in the first few weeks of life; however, pathological jaundice is associated with an increased risk of long-term complications and mortality only a few studies have been conducted on the determinants of neonatal jaundice in Ethiopia. The aim of this study was to identify the determinants of neonatal jaundice (pathological) among neonates admitted to neonatal intensive care units in Sidama Region general and referral public hospitals.

**Methods:**

a hospital-based unmatched case-control study was conducted among 270 neonates in public hospitals of Sidama Region from June 23 to August 8, 2022. We used a pre-tested interviewer-administered questionnaire and collected by open data kit (ODK) then the data was downloaded and exported to Microsoft Excel worksheets (XLS) and imported to SPSS version 26 for further analysis. Bi-variable logistic regression analysis was performed. Variables with a P-Value of less than 0.25 were included in multivariable logistic regression. Multi-variable logistic regression was performed and Adjusted Odds ratio (AOR) with a 95% confidence interval was computed and statistical significance was declared at a p-value <0.05.

**Results:**

a total of 270 neonates with mothers (90 cases and 180 controls) with a response rate of 100% were included in this study. Factors significantly increased the odds of developing neonatal jaundice were multiparty (AOR=2.869(95%CI 1.426-5.769)), prolonged duration of labor (AOR=4.618(95%CI 1.689 - 12.625)). ABO incompatibility (AOR=3.362(95%CI, 1.185 - 9.537)). Preterm (AOR=2.936(95%CI, 1.2456.923)), birthasphyxia (AOR=2.278(95%CI,1.1454.531)) and polycythemia (AOR=3.397(95%CI, 1.147-10.061)).

**Conclusion:**

in this study multiparty, prolonged duration labor, ABO incompatibility, preterm gestational age, birth asphyxia, and polycythemia were factors that significantly increased the odds of developing neonatal jaundice.

## Introduction

Jaundice is one of the neonatal illnesses that require medical treatment during the neonatal period [1]. It occurs in most neonates as “physiological” or “normal” jaundice in the first week of life with clinical manifestation of yellowish discoloration of the skin, mucous membranes, or sclera caused by bilirubin deposition [2-4]. Jaundice is clinically detected when the Total Serum Bilirubin (TSB) concentration is greater than or equal to 5 mg/dL (85mmol/liter) in newborns [2,5]. Pathological jaundice increases the risk of short and long-term complications including hospitalization costs, hearing loss, kernicterus risks, and mortality [6]. Hyperbilirubinemia in newborns is linked to several health problems and when it is severe the risk of developing a permanent neurological malfunction increases [7].

In 2016, a global child mortality report showed that neonatal jaundice accounted for 1309 deaths per 100000 live births and ranked seventh globally among all causes of neonatal deaths in the early-neonatal period (0-6 days), whereas in sub-Saharan Africa (SSA) it is the eighth leading cause of neonatal mortality [8]. In the late-neonatal period (7-27 days), jaundice accounted for 187 deaths per 100000 and ranked ninth globally, while the disease ranked 12^th^ in SSA [8].

One of the complications of Neonatal Jaundice (NNJ) is kernicterus and studies show that the prevalence of kernicterus in Low-Middle Income Countries (LMICs) like SSA, Latin America, Eastern Europe/Central Asia, and South Asia regions was estimated as high as 73/100,000 in comparison to 10/100,000 live births in high-income countries [9]. More than 83% of survivors with kernicterus had ended with one or more impairments [10].

According to the Ethiopian demographic health survey of 2016 and 2019, the rate of neonatal mortality had increased from 29 per 1000 live births to 30 per 1000 live births, with neonatal jaundice being one of the primary causes [11,12].

Neonatal jaundice is one of the concerns for different countries of the world because neonatal hyperbilirubinemia is linked to both fatal and non-fatal health outcomes worldwide and also with some survivors experiencing long-term complications [13]. The World Health Organization (WHO) Sustainable Development Goals (SDG) target that by 2030, the global average neonatal mortality rate (NMR) should be <12/1,000 live births, and every country should reduce its neonatal mortality rate by at least two-thirds from the 2010 baseline [14]. Ethiopian health sector transformation plan (HSTP II) to decrease neonatal mortality from 33 per 1,000 live births to 21 per 1,000 live births from 2020/21 to 2024/25 [15].

Despite the above efforts, Ethiopia is one of the countries with higher neonatal mortality, and neonatal jaundice is among the leading causes of neonatal mortality in the country. Additionally, neonatal jaundice results in a high burden for healthcare services worldwide, especially in low-income and middle-income countries like SSA, including Ethiopia [16]. To attain the target of the SDG and HSTP II plan in our country, working on preventable causes of neonatal mortality like neonatal jaundice is very important, and understanding the contributing factors for the development of neonatal jaundice is very crucial. Even though neonatal jaundice is among the leading causes of neonatal mortality, only a few studies have been conducted on the determinants of neonatal jaundice. Therefore, this study aimed to identify the factors that determine the occurrence of neonatal jaundice in Sidama region general and referral public hospitals by an added variable not included in the previous studies like syphilis during pregnancy.

## Methods

**Study design and period:** a hospital-based unmatched case-control study was conducted in public hospitals of Sidama region from June 23, 2022, to August 8, 2022.

**Study setting:** Sidama national regional state is one of 11 regions found in Ethiopia and Hawassa City is the capital of the region which is located 273 km south of Addis Ababa (A.A.). The region is bordered in the north and east by Oromia and in the south and west by Oromia and the south region. According to the 2022 Ethiopian Central Statistical Agency report estimation, the total population of the region was 4,623,000 including Hawassa City administrations [17].

**Participants:** all neonates with any neonatal cases admitted to the Neonatal Intensive Care Unit (NICU) in Sidama region general and referral public hospitals in the neonatal period.

### Study population

**Cases:** all neonates who diagnose neonatal jaundice (pathological jaundice) based on clinical sign and symptoms, history, and laboratory investigation and confirmed by pediatricians, residents, and general practitioners (GPs) among neonates admitted to the NICU in Sidama region general and referral public hospitals during the study period.

**Control:** all selected neonates who diagnose without neonatal jaundice (pathological) by pediatricians, residents, and GPs among neonates admitted to NICU in Sidama region general and referral public hospitals during the study period.

**Inclusion and exclusion criteria:** all neonates admitted to NICU with a confirmed diagnosis of neonatal jaundice (pathological) were considered cases during the study period and all neonates admitted to NICU without neonatal jaundice (pathological) during the study period were included. Meanwhile, neonates with and without neonatal jaundice (pathological) who were abandoned neonates, neonates whose mothers were critically ill during the study period, incomplete laboratory investigation and charts were excluded and also in neonates with NNJ those coming for the second time with similar diagnosis (readmitted neonate with pathological jaundice) and neonates without NNJ those coming for the second time (readmitted neonates with any diagnosis) during the study period were excluded from the study.

**Sample size determination:** the sample size was calculated by using the Open EPI INFO 7.2 STAC CALC double population proportion formula. For each factor's power of 80%, a confidence level of 95% and a 2: 1 control-to-case ratio was considered. Proportion of exposure among control groups (P2)=11.1% and AOR =2.88 taken [18] and by adding 10% non-response the final sample size was 270 (90 cases and 180 controls).

**Sampling techniques and procedure:** all general and referral public hospitals in the Sidama region were selected for the study. All neonates from the five public hospitals admitted to NICU during the study period were considered to be study participants. All neonates diagnosed with neonatal jaundice (pathological) during the data collection period in each hospital were consecutively selected as a case. The controls were selected following the case of neonates who have not developed neonatal jaundice by simple random sampling techniques.

**Variables and operational definitions:** neonatal jaundice is a yellowish discoloration of the skin, mucous membranes, and sclera caused by increased bilirubin in the blood.

**Cases:** neonates diagnosed as jaundiced (pathological jaundice) through history, clinical sign and symptoms, and or laboratory investigation (jaundice appears in first 24 hrs, or serum bilirubin is >12mg/dl in full-term infants and >15mg/dl in preterm or prolonged jaundice after two weeks for term and three weeks for preterm) diagnosed and confirmed by physicians (pediatricians, residents and general practitioners) [2,3].

**Controls:** neonates without the diagnosis of neonatal jaundice are considered as controls.

**Polycythemia:** is defined as peripheral venous blood of HCT >65%, or a capillary blood sample is higher by 10 - 15% than venous blood [3].

**Data collection tools and procedure:** the data collection tool was developed from a literature review in previous studies conducted in different countries based on the factors reviewed and prepared by the English version, then translated to local language Sidama Affo. The data was collected using a pre-tested interview administered structured questionnaire with ODK (open data kit). The tools contain questions that assessed socio-demographic characteristics, maternal medical characteristics, obstetrics characteristics, and neonatal characteristics. To collect the data, three nurses and two midwives who were fluent in the local language were recruited as data collectors, while two Master of Public Health (MPH)/Reproductive Health (RH) were recruited for supervising and coordination.

**Data quality assurance:** to keep the quality of the data, the English version of the questionnaire was translated into the local language Sidaamu Affo, and then back to English to maintain its consistency for actual data collection purposes. Data collectors and supervisors were given detailed training by the principal investigator for one day on the objectives of the study. Before the actual data collection, a pre-test was carried out on 5% of the sample size at the other hospital (Wolayita Sodo Referral Hospital), and based on the findings of the pretest; possible amendments were made to the questionnaire.

**Data processing and analysis:** the collected data were checked for consistency and completeness, downloaded from the Kobo toolbox user server, exported to XLS (SPSS) then download. Finally, the file was imported to SPSS Version 26 for further analysis. The descriptive statistics, such as frequency and percentage, were performed to describe study population. Bi-variable logistic regression analysis was done to the crude relationship between the dependent and each independent variable and to select candidate variables for multiple logistic regressions. The fitness of logistic regression models was assessed using the Hosmer-Lemeshow test. Variables with bi-variable logistic regression P-value less than 0.25 were included in multi-variable logistic regression. Multi-variable logistic regression was performed to identify independent predictors of neonatal jaundice. Statistically, the significance was declared at a P-value of <0.05. Finally, to present the result text and tables were used.

**Ethical approval and consent to participants:** ethical clearance was obtained from the institutional review board (IRB Ref.No;IRB/196/14) of Hawassa University College of Medicine and Health Sciences. Clearance and an official supporting letter were obtained from the Department of Midwifery and submitted to the Sidama Region Health Office. A supporting letter was also taken from the Sidama Region Public Health Institute and given to the medical directors of each hospital. Written informed consent was obtained from the study participants (the neonates' mothers) after explaining the importance of the study, and the privacy and confidentiality of their information were maintained throughout the study. In accordance with the Helsinki Declaration, all relevant ethical principles were followed and respected.

## Results

**Socio-demographic characteristics:** a total of two hundred seventy neonates with mothers (90 cases, diagnose with neonatal jaundice and 180 controls without a diagnosis of neonatal jaundice) with a response rate of 100% were included in this study. In the majority of the cases, 63 (70%) neonates were male and 27 (30%) of the cases neonates were female. Nearly two-thirds of the neonates in cases 59 (65.55%) and in control 99 (55.00%) were in the age of fewer than two days during admission to NICU and the median age of neonates was 2 days with (IQR 1- 5 days ) and also the median age of the mother was 28 years (IQR 25-30-year-old) (Table 1).

**Table 1:** socio-demographic characteristics of mother and neonate in Sidama region general and referral public hospitals, Sidama, Ethiopia, 2022

Variables	Category	Neonatal jaundice status		
		Cases N (%)	Controls N (%)	Total
Sex of neonate	Female	27(30.00%)	75(41.67%)	102(37.78%)
	Male	63(70.00%)	105(58.33%)	168 (62.22%)
Age of neonate	<2 days	59(65.55%)	99(55.00%)	158(58.52%)
	2-7 days	24(26.67%)	42(23.33%)	66(24.44%)
	>7 days	7(7.78%)	39(21.67%)	46(17.04%)
Age of mother	<20	7 (7.78%)	9(5.00%)	16(5.93%)
	20-35	75(83.33%)	158(87.78%)	233(86.30%)
	>35	8(8.89%)	13(7.22%)	22(8.14%)
Educational status of a mother	No formal education	32(35.56%)	55(30.56%)	87(32.22%)
	Primary	30(33.33%)	63(35.00%)	93(34.44%)
	Secondary	9(10.00%)	42(23.33%)	51(18.89%)
	Collage and above	19(21.11%)	20(11.11%)	39(21.67%)

**Medical and obstetrics characteristics of mothers:** mothers whose neonates were included in this study were asked about some medical conditions. Five (5.56%) of mothers in cases and 14 (7.78%) in controls had diabetic mellitus. The majority of neonates in cases 64 (71.1%) and 99 (55%) in controls were delivered from multipara mothers. Among mothers who gave alive birth before these 7 (10.94%) cases and 11 (11.11%) of controls had a previous history of a baby with neonatal jaundice (Table 2).

**Table 2:** medical and obstetrics disorders characteristics of mothers in Sidama region referral and general public hospitals, Sidama, Ethiopia, 2022

Variables	Category	Neonatal jaundice status		
		Cases N (%)	Controls N (%)	Total
DM	Yes	5(5.56%)	14(7.78%)	19(7.04%)
	No	85(84.44%)	166(92.22%)	251(92.96%)
Anemia	Yes	10(11.11%)	15(8.33%)	25(9.26%)
	No	80(88.89%)	165(91.67%)	245(90.74%)
Syphilis during pregnancy	Yes	10(11.11%)	13(7.22%)	23(8.52%)
	No	79(87.78%)	160(88.89%)	239(88.52%)
	Unknown	1(1.11%)	7(3.89%)	8(2.96%)
Maternal parity	Primipara	26 (28.89%)	81 (45.00%)	107 (39.63%)
	Multipara	64 (71.11%)	99(55.00%)	163(60.37%)
Hx previous baby developed NJ (n=163)	Yes	7(10.94%)	11 (11.11%)	18(11.04%)
	No	57(89.06%)	88(88.89%)	145(88.96%)
Mode of delivery	Spontaneous	49(54.44%)	107(59.44%)	156(57.78%)
	Cesarean section	35(38.89%)	66(36.67%)	101(37.41%)
	Instrumental	6(6.67%)	7(3.89%)	13(4.81%)
Onset of labor	Spontaneous	74((82.22%)	153(85%)	227(84.07%)
	Induced with oxytocin	6(6.67%)	11(6.11%)	17(6.30%)
	Not in labor	10(11.11%)	16(8.89%)	26(9.63%)
Duration of labor	Prolonged	15(15.67%)	11(6.11%)	26(9.67%)
	Normal	75(83.33%)	169(93.89%)	244(90.37%)
Obstetrics complication	Yes	28(31.11%)	30(16.67%)	58(21.48%)
	No	62 (68.89%)	150(83.33%)	212(78.52%)

DM: diabetes mellitus; NJ: neonatal jaundice

**Neonatal characteristics:** more than half of neonates in cases 48 (53.33%) and 48 (26.67%) in controls were low birth weight and also the gestational age of neonates at birth in cases 50 (55.56%) and in controls, 42 (23.33%) were preterm neonates. More than one-third of the neonates in cases 34 (37.78%) and around one-fourth of 41 (22.78%) of controls had birth asphyxia (Table 3).

**Table 3:** neonatal characteristics neonates admitted in NICU in Sidama region referral and general hospitals 2022

Variables	Category	Neonatal jaundice status		
		Cases N(%)	Controls N(%)	Total
Birth weight	<2500 gm	48(53.33%)	48(26.67%)	96(35.56%)
	2500-4000gm	40(44.45%)	110(61.11%	150(55.55%)
	>=4000 gm	2(2.22%)	22(12.22%	24(8.89%)
Gestational age at birth	Preterm	50(55.56%)	42(23.33%)	92(34.07%)
	Term	40(44.44%)	138(76.67%)	178(65.93%)
Birth asphyxia	Yes	34(37.78%)	41(22.78%)	75(27.78%)
	No	56(62.22%)	139(77.22%)	195(72.22%)
Polycythemia	Yes	14(15.56%)	9(5.00%)	23(8.52%)
	No	76(84.44%)	171(95.00%)	247(94.48%)
Neonatal sepsis	Yes	49(54.44%)	70(38.89%)	119(44.07%)
	No	41(45.56%)	110(61.11%)	151(55.93%)
Birth trauma	Yes	11(12.22%)	12(6.67%)	23(8.52%)
	No	79(87.78%)	168(93.33%)	247(91.48%)
Feeding methods	EBF	77(85.56%)	150(83.33%)	227(84.07%)
	Formula	4(4.44%)	10(5.56%)	14(5.19%)
	Mixed	4(4.44%)	14(7.78%)	18(6.67%)
	Others	5(5.56%)	6(3.33%)	11(4.07%)
Feeding frequency	2-3 hours	64(71.11%)	125(69.44%)	189(70.00%)
	> 3 hours interval	10(11.11%)	27(15.00%)	37(13.70%)
	Demand	16(17.78%)	28(15.56%)	44(16.30%)
Hypothermia	Yes	46(51.11%)	57(31.67%)	103(38.15%)
	No	44(48.89%)	123(68.33%)	167(61.85%)
Hypoglycemia	Yes	9(10.00%)	27(15.00%)	36(13.33%)
	No	81(90.00%)	153(85.00%)	234(86.67%)

EBF: exclusive breast feeding

**Bi-variable and multivariable binary logistic regression model:** bi-variable logistic regression was computed for each variable and variables with P-value <0.25 were taken into a multivariable logistic regression model. The candidate variables for multivariable logistic regression were the educational status of mothers, maternal parity, and duration of labor, obstetrics complication, ABO incompatibility, gestational age, birth asphyxia, polycythemia, neonatal sepsis, and neonatal hypothermia (Table 4).

**Table 4:** bi variable and multivariable logistic regression model for the determinants of neonatal jaundice in Sidama region general and referral public hospitals, Sidama, Ethiopia, 2022

Variables	Category	Neonatal jaundice status		OR (95% CI)	
		Cases	Controls	COR 95%CI	AOR 95%CI
Educational status of the mother	No formal education	32	55	0.61(0.28-1.32)	0.54(0.21-1.41)
	Primary	30	63	0.50(0.23- 1.08)	0.56(0.21-1.46)
	Secondary	9	42	0.23(0.09-0.59)	0.35 (0.113-1.09)
	Above	19	20	1	1
Maternal parity	Primipara	26	81	1	1
	Multipara	64	99	2.01(1.17-3.46)	2.87(1.43-5.77)*
Duration of labor	Prolonged	15	11	3.07(1.35-7.1)	4.62(1.69-12.63)*
	Normal	75	169	1	1
ABO incompatibility	Yes	16	10	3.57(1.55-8.3)	3.36(1.18- 9.54)*
	No	73	163	1	1
Obstetrics complication	Yes	28	30	2.26(1.25-4.1)	1.48(0.66 -3.33)
	No	62	150	1	1
Gestational age at birth	Preterm	50	42	4.12(2.39- 7.05)	2.94(1.25-6.92)*
	Term	40	138	1	1
Birth asphyxia	Yes	34	41	2.06 (1.19-3.57)	2.28 (1.15-4.53)*
	No	56	139	1	1
Polycythemia	Yes	14	9	3.5(1.45-8.44)	3.4(1.15-10.1)*
	No	76	171	1	1
Neonatal sepsis	Yes	49	70	1.9(1.13- 3.1)	1.29(0.66- 2.5)
	No	41	110	1	1
Hypothermia	Yes	46	57	2.26(1.3-3.8)	1.61(0.81-3.20)
	No	44	123	1	1

ABO: blood group A, B, and O; *: significant at p-value of <0.05

## Discussion

This unmatched case-control study was conducted in Sidama region general and referral public hospitals and aimed to identity the determinants of neonatal jaundice. Parity of mothers, prolonged duration of labor, ABO incompatibility, preterm gestational age, birth asphyxia, and polycythemia were the identified determinants of neonatal jaundice.

In this study maternal parity was determined the occurrence of neonatal jaundice. The odds of neonates who were delivered from multipara mothers were 2.87 times higher among neonates with neonatal jaundice as compared to neonates without neonatal jaundice. This finding disagrees with the study conducted in south India [19] and Swedish [20] since both studies revealed that the odds of neonatal jaundice are higher in neonates who delivered from primipara mothers than neonates delivered from multipara. The possible explanation for this is due to an increased number parity the women exposed to different risk factors like preterm delivery and other obstetrics complication that might lead to neonatal jaundice. A study shows that the risks of any obstetric complications, neonatal morbidity, and perinatal death increased from parity 4 or 5 therefore in current study those mothers included as multipara [21].

The odds of prolonged duration of labor are 4.62 times higher among neonates with neonatal jaundice than neonates without neonatal jaundice. This finding is supported by the studies conducted in Tehran, Iran [22], Amhara [18] and Tigray, Ethiopia [23]. This might be due to as labor prolongs the newborn may be exposed to different problems like scalp injure (cephalhematoma) these conditions are known predictors of neonatal jaundice by increasing bilirubin production [1,24].

This study showed that the odds of ABO incompatibility were 3.36 times higher among neonates with neonatal jaundice than neonates without neonatal jaundice. This finding is supported by the studies conducted in North India [25], South India [19], multi-centered prospective study [26], and in Tigray region, Ethiopia [23] and also recently published systematic and meta-analysis study in chain [27]. This might be due to the mother's red blood cells might cross into the placenta or fetus during pregnancy and might develop antibodies that can attack the newborn's blood cells and cause the baby to start to break down extra red blood cells even before born and results neonatal jaundice.

This study also revealed that the odds of preterm neonates 2.94 times higher in neonates with neonatal jaundice than in neonates without neonatal jaundice. This finding agreed with the studies conducted in Ghana [28], South India [19], and Kerala, India [29]. This might be due to preterm neonates prone to hepatic immaturity, reduction in the uptake and conjugation of bilirubin, and increased enter the hepatic circulation of bilirubin due to intestinal immaturity and delayed enteral feeding, then final the unconjugated bilirubin clearance decreased and results in the occurrence of neonatal jaundice [24].

Another finding of this study was the odds of birth asphyxia were 2.28 times higher among neonates with neonatal jaundice than neonates without neonatal jaundice. This finding agreed with the studies in Amhara [18] and Tigray [30]. This might be due to birth asphyxia leading to low oxygen (hypoxic) that may cause damage or reduces the activity of the transferee´s enzyme and due to hypoxemia; different organ systems of the body are affected and resulting in increased levels of serum bilirubin. Since the occurrence of birth asphyxia is directly related to antepartum, intrapartum, and immediate post-partum conditions. Especially during intrapartum it is mostly significantly associated with obstructed labor and prolonged labor they also determinate neonatal jaundice. In this study, prolonged duration of labor was revealed as a determinant of neonatal jaundice [31]. This finding contradicts the study conducted in a multi-centered prospective, cross-sectional, observational study in preterm neonates that showed the odds of birth asphyxia at 0.52 time decrease the occurrence of neonatal jaundice [26]. The possible reason might be due to the different source populations because the current study includes all neonates during the neonatal period as source populations regardless of gestational age.

This study revealed that neonates with neonatal jaundice were 3.4 times more likely to have polycythemia than neonates without neonatal jaundice. The finding of this study was supported by the study in Mekelle Tigray, Ethiopia [30]. This is due to an increased red blood cell (RBC) that results in elevated production of bilirubin then which leads to an increase in the load of bilirubin to be metabolized by the liver and final that results in an increase in the total serum bilirubin and elevated unconjugated bilirubin [24]. The published data showed that neonatal jaundice is the main presentation among neonates with polycythemia [32].

**Limitation of the study:** some medical characteristics of a mother were asked that were not recorded on medical charts and this might be introduced recall bias, and also another limitation of this study was during study period, in some hospitals there was a shortage on availability of laboratory investigation for serum bilirubin determination.

## Conclusion

In this study, multiparty, prolonged labor, ABO incompatibility, preterm gestational age, birth asphyxia, and polycythemia were factors that significantly increased the odds of developing neonatal jaundice. In this study, the identified determinants of neonatal jaundice were directly related to antepartum intrapartum, and postpartum-related conditions. To minimize these determining factors regional health offices and hospitals have to encourage and follow health care providers for early detection and risk assessment during those periods.

### What is known about this topic

- *Neonatal jaundice results in a high burden for healthcare services worldwide, especially in low-income and middle-income countries like sub-Saharan Africa, including Ethiopia;*
- *In Ethiopia, also different studies show that the prevalence of neonatal jaundice ranges from 13.3% to 37.3% but they are not showing the prevalence of pathological jaundice;*
- *Even though previous studies were conducted on neonatal jaundice but on determinates neonatal jaundice (pathological) with actual cases based (prospective case-control) studies not done in Southern Ethiopia*.

### What this study adds

- *Multiparty, prolonged duration labor, ABO incompatibility, preterm gestational age, birth asphyxia, and polycythemia were factors that significantly increased the odds of developing neonatal jaundice;*
- *The identified determinants of neonatal jaundice were directly related to antepartum intrapartum, and postpartum-related conditions therefore to minimize these determining factors early detection and risk assessment during those periods is important;*
- *Identifying the risk factors of neonatal jaundice is important to minimize the long and short-term complications*.
